# Nocturnal polygraphy in neuromuscular disorders: is it a useful diagnostic tool?

**DOI:** 10.1055/s-0046-1825767

**Published:** 2026-08-13

**Authors:** Aleciane Cristine de Oliveira, Thayanne dos Santos Corrêa, Lucas Figueira Vieira, Iago Resende Carvalho, Daniela Name Chaul, Ana Elizabeth Cunha Guimarães de Almeida, Vitor Laguardia Guido Faria, Paulo Cézar Simamoto Júnior, José Fausto de Morais, Fernando Gustavo Stelzer, Isabela Maria Bernardes Goulart, Diogo Fernandes dos Santos

**Affiliations:** 1 Universidade Federal de Uberlândia, Hospital das Clínicas, Programa de Pós-Graduação em Ciências da Saúde, Uberlândia MG, Brazil.; 2 Universidade Federal de Uberlândia, Hospital das Clínicas, Ambulatório de Doenças Neuromusculares, Unidade de Atenção Domiciliar e Cuidados Paliativos, Uberlândia MG, Brazil.; 3 Universidade Federal de Uberlândia, Faculdade de Medicina, Uberlândia MG, Brazil.; 4 Universidade Federal de Uberlândia, Centro de Ciências Biomédicas, Departamento de Biociências, Uberlândia MG, Brazil.; 5 Universidade Federal de Uberlândia, Programa de Pós-Graduação em Odontologia, Uberlândia MG, Brazil.; 6 Universidade Federal de Uberlândia, Instituto de Matemática e Estatística, Uberlândia MG, Brazil.; 7 Universidade de São Paulo, Faculdade de Medicina de Ribeirão Preto, Departamento de Neurologia e Ciências do Comportamento, Ribeirão Preto SP, Brazil.; 8 Universidade Federal de Uberlândia, Hospital das Clínicas, Centro de Referência Nacional em Hanseníase e Dermatologia Sanitária, Laboratório de Biologia Molecular e Biotecnologia, Uberlândia MG, Brazil.

**Keywords:** Neuromuscular Diseases, Sleep Apnea, Obstructive, Polysomnography

## Abstract

**Background:**

Patients with neuromuscular diseases (NMDs) have a high incidence of sleep-related breathing disorders. These include obstructive sleep apnea (OSA), which is characterized by recurrent episodes of upper airway obstruction during sleep, resulting in intermittent hypoxemia and sleep fragmentation. Obstructive sleep apnea may be more common in patients with NMDs than in the general population.

**Objective:**

To assess the prevalence of OSA through home polygraphy at a public university hospital during respiratory evaluations of patients with NMDs, and its contribution to the indication of non-invasive ventilation. It did not aim to validate a model for early indication.

**Methods:**

The present prospective study collected data over the period from May 2021 to June 2024, using medical records and tests such as spirometry to evaluate pulmonary respiratory function, home polygraphy, and sleep assessments using the Epworth Sleepiness Scale (ESS) and a symptom questionnaire.

**Results:**

A total of 74 patients were included, 64.7% (n = 48) of whom had OSA. The most prevalent NMDs were muscular dystrophy (n = 22; 29.7%), myasthenia gravis (n = 13; 17.6%), and amyotrophic lateral sclerosis (ALS) (n = 11; 14.9%). Apnea-hypopnea index (AHI) values were higher in the supine position (11.6 ± 12.6) compared to the AHI in the non-supine position (7.0 ± 14.1), which indicates, on average, a mild degree of OSA. The ESS revealed that 40.5% of patients had scores indicating excessive daytime sleepiness (EDS).

**Conclusion:**

The findings reinforce the importance of early OSA diagnosis in patients with NMDs.

## INTRODUCTION

Neuromuscular diseases (NMDs) encompass a group of disorders that affect upper and lower motor neurons, peripheral nerves (including hereditary and acquired neuropathies), the neuromuscular junction (such as myasthenia gravis and congenital myasthenia), and/or muscles (including myopathies and dystrophies). 1 2 3 4 5 They are characterized by progressive muscle weakness—often severe proximal weakness, muscle atrophy, and wasting—leading to loss of ambulation, major gait impairment, contractures, and progressive scoliosis; prominent bulbar impairment causes dysphagia, dysarthria, and recurrent aspiration, while respiratory muscle compromise manifests as nocturnal hypoventilation, orthopnea, and respiratory failure. 1 2 3 4 5 Most NMDs are considered rare, with a prevalence of less than 50 per 100,000. 6

Restrictive respiratory impairment reflects diminished lung expansion from reduced respiratory system compliance or inspiratory capacity, with hallmark reductions in lung volumes—principally total lung capacity (TLC) and forced vital capacity (FVC). In NMDs, restriction is predominantly extrinsic, secondary to respiratory muscle weakness that limits diaphragmatic descent and chest wall excursion, producing proportional declines in TLC and FVC, reduced maximal inspiratory and expiratory pressures, diminished sniff nasal pressure and peak cough flow, and progressive nocturnal hypoventilation with hypercapnia. 7 8 Clinically significant sequelae include exertional dyspnea, orthopnea, ineffective cough with retained secretions, and recurrent atelectasis, which mandate periodic lung–volume and respiratory–muscle testing, nocturnal oximetry, and early consideration of noninvasive ventilatory support. 1

Patients with NMDs also have a high incidence of sleep-related breathing disorders (SRBDs), 9 including obstructive sleep apnea (OSA), central sleep apnea (CSA), and nocturnal hypoventilation (NH). Due to the association of these disorders with typical restrictive respiratory impairment, both OSA and NH are frequent in these patients. 2 6 Additionally, studies suggest that OSA may be more prevalent in individuals with NMDs than in the general population. 6 9 10

Obstructive sleep apnea is characterized by recurrent episodes of airway obstruction during sleep, leading to intermittent hypoxemia and sleep fragmentation, with resulting daytime dysfunction, including excessive daytime sleepiness. 11 This condition poses a public health concern and is linked to a reduction in quality of life. 12 In he general population, OSA affects 3 to 7% of men and 2 to 5% of women. Brazil ranks among the 10 countries with the highest estimated number of individuals with OSA, with 49 million people having an apnea-Hypopnea Index (AHI) ≥ 5 events per hour, and 25 million with AHI ≥ 15 events per hour. 13 The prevalence of OSA is high in different NMDs, reaching up to 70% in Duchenne muscular dystrophy (DMD), 14 37 to 66% in myotonic dystrophy type 1 (DM1), 15 and approximately 36% in myasthenia gravis (MG). 16 In amyotrophic lateral sclerosis (ALS), the occurrence is less consistent. Still, OSA events may also be present, 17 reinforcing the need for evaluation in all patients with NMD who present sleep-related symptoms. 6

Type-I polysomnography (PSG) is considered the gold standard for diagnosing and stratifying the severity of OSA. However, it is a test that requires a sleep laboratory and trained technical personnel, has inherent costs, and is not widely available. 18 Although PSG is mandatory in many countries, its application faces challenges due to limited availability, resulting in delayed diagnoses and potential missed opportunities for treatment. Therefore, it is essential to provide alternative strategies that ensure OSA identification. 19 One such alternative is the home-sleep apnea test (HSAT), or home polygraphy (HP), used in OSA screening, which simultaneously monitors oxygen saturation, nasal airflow, snoring, heart rate, respiratory effort, and body position using surface sensors. 20 In addition to being widely accessible, this test can be easily performed at home, facilitating OSA screening. 19 Thus, the present study aimed to assess the prevalence of OSA through HP in a public university hospital in Brazil during the respiratory evaluation of patients with NMDs.

## METHODS

### Study description

The present was a prospective and observational analysis conducted with patients diagnosed with NMDs, monitored at a specialized NMDs outpatient clinic of a public university hospital. Patients with a diagnosis of NMD who were already routinely evaluated for restrictive pulmonary impairment and indication for non-invasive ventilation (NIV) were included, as well as those with NMDs who already had an FVC below 70% and a recommendation for home NIV. The exclusion criteria are tracheostomized patients.

The study was previously approved by the Research Ethics Committee involving Human Subjects of Universidade Federal de Uberlândia (UFU) under opinion number 44467320.4.0000.5152, in 2021. All participants were adequately informed about the study's objectives and procedures and subsequently signed the Informed Consent Form (ICF), in accordance with ethical and legal standards.

### Data collection

Data collection took place from May 2021 to June 2024. Demographic information (age, sex), clinical data (NMD type), and results of pulmonary function tests (spirometry) were extracted from medical records.

### Spirometry

Spirometry was performed to assess pulmonary respiratory function. It consists of an inhalation followed by a forced exhalation to evaluate, among several variables, particularly the forced vital capacity (FVC), defined as the total volume forcefully exhaled. 21 Patients with NMDs have a tendency towards reduced FVC in the supine position compared to the seated position due to the gravitational effect amidst diaphragmatic neuromuscular weakness. 22 Therefore, spirometry was performed to assess FVC in NMD patients in two conditions: seated (pre-FVC) and lying position (post-FVC), to investigate the correlation with OSA prevalence. All patients with an FVC of less than 70% were recommended to use home NIV. For statistical purposes, the seated position was adopted as a reference.

Based on the spirometry results, the study population was divided into 2 subgroups: the 1st included patients with FVC values below 70% (n = 23; 42.59%), and the 2nd comprised patients with FVC values above 70% (n = 31; 57.4%), for a more detailed analysis.

### Home polygraphy

For HP, prior scheduling was arranged with the patient to deliver the portable ApneaLink Air device (ResMed Inc.). At that time, participants received detailed instructions regarding the exam's objectives, the physiological parameters to be monitored, and the importance of correct sensor placement and strict adherence to usage instructions. During the visit, a practical demonstration of device handling was conducted, and any questions or doubts were clarified. Equipment return was scheduled for the following day. Data were collected and subsequently extracted using the ResMed AirView (ResMed) software, analyzed by a specialized physician according to the guidelines of the American Academy of Sleep Medicine (AASM) 2012. 23 The exam's quality was evaluated based on a minimum recording time of 4 hours of sleep; examinations yielding values below this threshold were repeated. Variables extracted from valid records included: apnea index (AI), hypopnea index (HI), AHI in supine and non-supine positions, obstructive apnea index (OAI), central and mixed apnea, and oxygen desaturation index (ODI).

Hypopnea was characterized as a reduction of ≥ 30% in the nasal airflow for at least 10 seconds, associated with a drop in oxygen levels by ≥ 3% from the participant's baseline, as established by the AASM criteria.

### Epworth Sleepiness Scale (ESS)

Daytime sleepiness was assessed using the Epworth Sleepiness Scale (ESS), initially developed by Johns in 1991. 24 For the current study, the Brazilian Portuguese version validated by Bertolazi et al. 25 was used. The ESS is composed of eight items that assess an individual's propensity to fall asleep in various everyday situations, such as: sitting and reading, watching television, sitting inactive in a public place, being a passenger in a car for an hour without a break, lying down to rest in the afternoon, sitting and talking to someone, sitting quietly after lunch without alcohol, and in a car stopped in traffic for a few minutes. The total score ranges from 0 to 24, with scores above 10 indicating excessive daytime sleepiness (EDS), which may be mild, moderate, or severe. In patients with severe dysarthria and/or communication impairment by NMD, the scale was not applied.

### Sleep-related symptom questionnaire

Additionally, patients completed a questionnaire on sleep-related symptoms, including snoring, observed respiratory pauses, gasping during sleep, daytime sleepiness, morning headaches, sleep fragmentation, nocturia, concentration difficulties, memory loss, decreased libido, and irritability. Responses were recorded in a binary format (presence or absence of the symptom).

### Statistical analysis

The normality of the distribution of quantitative variables was assessed using the Shapiro-Wilk test due to the small sample size. For analysis, frequency distributions, means, and standard deviations were used for normally distributed quantitative variables, while frequency distributions were used for nominal or ordinal qualitative variables. To evaluate baseline O2 saturation and total AHI we used Spearman's rank correlation coefficient. For the ESS, the frequency distributions of responses for each item were constructed, and reliability was evaluated using item-total correlation and Cronbach's Alpha. Associations between two nominal qualitative variables were tested using the Chi-squared test with correction or Fisher's exact test, depending on the criteria outlined in the literature. Student's *t* -test, Welch's *t* -test, or the Mann-Whitney U test were used to compare means between groups with or without an indication for non-invasive ventilation devices. Data were analyzed using the statistical software IBM SPSS Statistics for Windows (IBM Corp.), version 21.2 and JAMOVI (The Jamovi Project) version 2.3.28. The significance level for all statistical tests was set at *p* ≤ 0.05.

## RESULTS

The sample showed a wide range of baseline characteristics among the 74 participants ( Table 1 ), with the majority (n = 39; 52%) being male, a mean age of 43.9 ± 20.3 years, and a mean Body Mass Index (BMI) of 25.9 ± 5.6, indicating a predominantly overweight (n = 26; 35.1%) and obese (n = 16; 21.6%) population. The most prevalent NMDs were muscular dystrophies (n = 22; 29.7%), MG (n = 13; 17.6%), and ALS (n = 11; 14.9%). One patient had spinocerebellar ataxia type 3 (SCA3), presenting with brainstem and cerebellar dysfunction, associated with diffuse lower motor neuron impairment. 26

**Table 1:** Characterization of 74 participants based on sociodemographic and clinical variables

Variables	n	%
**Male sex**	39	52.7
**Age**		
< 22	13	17.6
22–60	42	56.8
> 60	19	25.7
Mean ± SD		43.9 ± 20.3
**BMI**		
Underweight	7	9.5
Normal weight	23	31.1
Overweight	26	35.1
Obesity I	10	13.5
Obesity II	4	5.4
Obesity III	2	2.7
Did not respond*	2	2.7
Mean ± SD		25.9 ± 5.6
**Associated disease**		
1. Muscular dystrophy	22	29.7
2. Myasthenia Gravis	13	17.6
3. ALS	11	14.9
4. Acquired polyneuropathies	9	12.2
5. Myopathy	8	10.8
6. SMA	4	5.4
7. Hereditary polyneuropathies	4	5.4
8. Lambert-Eaton syndrome	2	2.7
9. Machado-Joseph disease	1	1.4

Abbreviations: ALS, amyotrophic lateral sclerosis; BMI, Body Mass Index; Lambert-Eaton syndrome, Lambert-Eaton myasthenic syndrome; n, number; SD, standard deviation; SMA, spinal muscular atrophy.

Note: *Lack of response was due to severe dysarthria and impaired communication resulting from neuromuscular disease.

Table 2 presents the results of the polygraphic indices and total ESS score. Forty-eight (64.7%) patients presented with OSA, characterized by an AHI of 10.6 (± 11.4). Most of the patients (44.6%; n = 33) had mild OSA, while a smaller proportion presented moderate (13.5%; n = 10) and severe OSA (6.8%; n = 5). The AHI in the supine position was 11.6 ± 12.6 events/hour, while in the non-supine position it was 7.0 ± 14.1 events/hour ( *p* = 0.04). The exam revealed an OAI of 1.3 ± 2.9, central apnea index of 0.6 ± 1.6, mixed apnea index of 0.1 ± 0.3, and an ODI of 23.4 ± 17.1, which was more significant than the total AHI 10.6 ± 11.4 ( *p* < 0.005).

**Table 2:** Characterization of the 74 participants according to their polygraphic indices and total Epworth Sleepiness Scale score

	n		%
**AHI – Total**			
Normal	26		35.1
Mild	33		44.6
Moderate	10		13.5
Severe	5		6.8
Mean ± SD		10.6 ± 11.4	
**AI**		2.5 ± 6.2	
**HI**		8.2 ± 6.7	
**AHI – supine**		11.6 ± 12.6	
**AHI – non-supine**		7.0 ± 14.1	
**OAI**		1.3 ± 2.9	
**OAI central**		0.6 ± 1.6	
**OAI mixed**		0.1 ± 0.3	
**ODI**		23.4 ± 17.1	
**FVC Pre (seated)**		76.1 ± 24.2	
**FVC Post (lying)**		70.7 ± 24.2	
**ESS**			
≤ 10	37		50.0
> 10	30		40.5
Did not respond	7		9.5
Mean ± SD		8.5 ± 6.1	

Abbreviations: AHI, apnea-hypopnea index; AI, apnea index; ESS, Epworth Sleepiness Scale; FVC, forced vital capacity; HI, hypopnea index; n, number; OAI, obstructive apnea index; ODI, oxygen desaturation index; SD, standard deviation.

Note: Only 54 patients were considered.

Spirometric values were obtained from 54 (73%) patients ( Table 3 ). The remaining 27 (17.5%) were unable to tolerate or cooperate with the procedure.

**Table 3:** Distribution of the 54 subjects who completed the exam according to the indication for ventilatory device use by variables of interest

Variables	FVC < 70 (n = 23)	%	FVC ≥ 70 (n = 31)	%	*p*
Male sex	11/23	47.8	17/31	54.8	0.815 ^2^
Age	46.9 ± 19.3		40.8 ± 18.1		0.214 ^3^
Baseline O2 saturation	97.1 ± 9.7		92.1 ± 17.3		0.175 ^3^
BMI	26.3 ± 5.3		25.5 ± 5.4		0.603 ^4^
ALS	4/23	17.4	3/31	9.7	0.671 ^2^
Myasthenia gravis	2/23	8.7	10/31	32.3	0.084 ^2^
Muscular dystrophy	10/23	43.5	5/31	16.1	0.056 ^2^
AHI – total	10.7 ± 10.9		8.6 ± 7.8		0.452 ^3^
AI – total	2.4 ± 5.4		1.1 ± 2.4		0.539 ^3^
HI – total	8.3 ± 6.5		7.5 ± 6.0		0.655 ^2^
AHI – supine	11.5 ± 12.7		9.5 ± 9.2		0.675 ^3^
AHI – non-supine	12.3 ± 21.6		5.9 ± 9.4		0.311 ^3^
OAI	1.3 ± 3.2		0.5 ± 0.9		0.563 _3_
Central apnea index	0.8 ± 1.8		0.5 ± 1.9		0.150 ^3^
Mixed apnea index	0.2 ± 0.5		0.0 ± 0.1		0.178 ^3^
ODI	19.2 ± 16.0		24.4 ± 15.6		0.234 ^3^
**FVC Pre (seated)**	**53.6 ± 15.6**		**92.8 ± 13.4**		**< .001** ^4^
**FVC Post (lying)**	**47.7 ± 14.0**		**87.7 ± 13.5**		**< .001** ^3^
**ESS**	**10.5 ± 6.3**		**5.9 ± 3.8**		**0.009** ^3^
Snoring	9/20	45.0	21/31	67.7	0.107 ^2^
Breathing pauses	2/20	10.0	10/31	32.3	0.136 ^2^
Gasping during sleep	3/20	15.0	13/31	41.9	0.086 ^2^
Daytime sleepiness	8/20	40.0	19/31	61.3	0.230 ^2^
Sleep fragmentation	8/20	40.0	17/31	54.8	0.454 ^2^
Morning headache	3/20	15.0	10/31	32.3	0.293 ^2^
Nocturia	12/20	60.0	20/31	64.5	0.977 ^2^
Difficulty concentrating	7/20	35.0	16/31	51.6	0.381 ^2^
Reduced libido	6/19	31.6	13/26	50.0	0.351 ^2^
Irritability	8/19	42.1	20/31	64.5	0.209 ^2^

Abbreviations: AHI, apnea-hypopnea index; AI, apnea index; BMI, Body Mass Index; ESS, Epworth Sleepiness Scale; FVC, forced vital capacity; HI, hypopnea index; O2, oxygen; OAI, obstructive apnea index; ODI, oxygen desaturation index.

Notes: 1. Corrected Chi-squared test; 2. Fisher's exact test; 3. Mann-Whitney U test; 4. Welch's *t* -test.

It was observed that 23 patients (47.8%) had reduced FVC (< 70%), while the majority of patients (n = 31; 54.8%) had normal FVC (≥ 70%). Forced vital capacity was decreased in the first group (FVC < 70%) in both seated (53.6 ± 15.6) and supine (47.7 ± 14.0) positions, compared to the other group (FVC > 70%) in the seated (92.8 ± 13.4) ( *p* < 0.001) and supine position (87.7 ± 13.5) ( *p* < 0.001). Regarding demographic characteristics, no differences were observed between the groups.

Patients with reduced FVC showed a higher prevalence of EDS, and ESS scores were significantly higher in patients with FVC < 70% (10.5 ± 6.3) compared to those with normal FVC (5.9 ± 3.8) ( *p* = 0.009). The AHI was similar between the groups, as were the other respiratory parameters evaluated in the HP. When comparing the AHI between patients stratified by FVC, it was found that, among those with reduced FVC (< 70% of predicted), OSA was classified as normal in 6 patients (26.0%), mild in 13 patients (56.5%), moderate in 1 patient (4.5%), and severe in 3 patients (13.0%). In contrast, among patients with preserved FVC, 13 patients (41.9%) had a normal AHI, 14 (45.2%) had mild OSA, 4 (12.9%) had moderate OSA, and none had severe OSA. Regarding symptoms, there were no statistically significant differences in respiratory symptom prevalence between the groups. No statistical difference was observed between patients with reduced or normal FVC in baseline O _2_ levels or total AHI ( *p* = 0.175).

## DISCUSSION

The relevance of OSA as a public health problem is highlighted by the significant associated comorbidities, including systemic arterial hypertension, myocardial infarction, stroke, and depression, in addition to a substantial impact on quality of life and cognitive performance. 18 However, the interaction between OSA and NMD represents a clinical and pathophysiological scenario of distinct complexity, which transcends the approach traditionally centered on upper airway obstruction.

Recognition and investigation of OSA may be undervalued in NMD patients in clinical practice. Previous evidence suggests that OSA prevalence may be higher in this population than in the general population, underscoring the need for complementary diagnostic approaches in these cases, as we observed in our results. 9

Similarly, in the study by Rodrigues Filho et al. (2021), the prevalence of OSA in 41 patients with NMD was 75.7%, with muscular dystrophy being the most common condition among the evaluated conditions. Furthermore, the authors demonstrated that the ODI performed better than other variables evaluated (e.g., AHI), reinforcing the possibility of using the ODI to identify OSA, as shown in our data, given that nocturnal oximetry is a more accessible and cost-effective alternative to polygraphy. 2 Saulnier et al. (2024) 9 evaluated 149 patients with slowly progressive NMD. In the present study, sleep apnea was primarily associated with central apnea (51.6%), unlike previous findings, which reported an obstructive predominance. The authors also observed that higher age and BMI were associated with OSA, suggesting additional relevant risk factors in this population.

Our study showed low values for obstructive, central, and mixed apneas, while the ODI was also significantly higher than the total AHI. The high ODI values observed indicate frequent and significant drops in oxygen levels during sleep, which can lead to cardiovascular, metabolic, and neuropsychiatric complications. Obstructive sleep apnea and NH often coexist in this group of patients, as observed. Relaxation of the throat muscles, a factor in OSA, is exacerbated by the intrinsic muscle weakness of NMDs, which can cause airway obstruction and lead to respiratory pauses, resulting in a consequent drop in oxygen saturation and hypercapnia. In addition, there is weakness of both inspiratory and expiratory muscles, which, in combination with reduced pulmonary compliance, results in increased respiratory effort and impaired deep inspiration, leading to chronic microatelectasis and ultimately a decline in vital capacity (VC). The pathophysiology of sleep-disordered breathing in NMD is, therefore, not a simple overlap of risk factors; it represents a complex interaction where the main problem is respiratory muscle failure. The therapeutic approach, therefore, should not be limited to the obstruction. 1 18 27

It is essential to note that the AHI was also higher in supine than in non-supine position, highlighting the impact of body position on respiratory function. When the patient lies on their back, total lung capacity is initially preserved, but VC can decrease significantly. This reduction is even more pronounced in individuals with diaphragmatic weakness, favoring early symptoms such as orthopnea and sleep disturbances. 22 In this context, due to progressive muscle weakness, most individuals become unable to engage in strenuous physical activities. Consequently, in most cases, dyspnea manifests only after disease progression. 1 However, follow-up through polysomnography enables earlier detection—while FVC values are still within the expected range—of respiratory muscle weakness, as indicated by parameters such as the AHI and ODI. 6

Overnight monitoring for sleep-disordered breathing ranges from screening tools to comprehensive diagnostic methods. Type-4 oximetry and capnography provide low-cost screening, with capnography improving detection of nocturnal hypoventilation in neuromuscular restrictive disease, though both lack precise event characterization. Type-3 respiratory polygraphy differentiates obstructive and central apneas by adding respiratory efforts and airflow to oximetry but may underestimate severity due to the absence of sleep staging, whereas polysomnography (types 1 or 2), particularly when extended with transcutaneous CO _2_ and respiratory electromyography, remains the reference standard. Saulnier et al. (2024) 9 indicate that isolated oximetry or arterial blood gases underestimate sleep disorders, whereas combined respiratory polygraphy, arterial blood gas analysis, and overnight capnography provide a more accurate diagnosis and guide optimal management of obstructive sleep apnea and alveolar hypoventilation. 9 19

The ESS revealed that a significant proportion of patients presented EDS. In agreement, the study by Hoffmann, Malo-Juvera, and Statland (2022), 28 which investigated self-reported sleep patterns in a large group of individuals with facioscapulohumeral muscular dystrophy (FSHD), identified a high prevalence of sleep disorders: of the 592 respondents, 66.2% reported reduced sleep quality and 15% had EDS. Complementarily, the review by Subramony et al. (2020), 29 which covered 3 decades of research in patients with myotonic dystrophy, found that EDS was present in 25 to 82% of studies evaluating unselected populations. Despite complaints of sleepiness, the mean ESS scores in most studies remained below the pathological cutoff of 10 points, suggesting that the scale may not fully capture the intensity of sleepiness reported by these patients. Considering that high ESS scores reflect a greater propensity to fall asleep in everyday situations—indicative of pathological or clinically relevant sleepiness— 30 this finding is particularly appropriate, given that EDS is associated with a higher risk of accidents, cognitive and functional impairments, and a significant reduction in quality of life.

The treatment of sleep-disordered breathing in patients with NMDs centers on positive airway pressure therapy. Although continuous positive airway pressure (CPAP) therapy is the first-line treatment for OSA in the general population, bilevel positive pressure ventilation (BiPAP) is often the modality of choice for patients with NMDs. It offers the advantage of differentiated inspiratory (IPAP) and expiratory (EPAP) pressures, which help relieve the strain on weakened respiratory muscles, more effectively correcting alveolar hypoventilation. In this context, CPAP is generally outweighed by BiPAP, particularly in the presence of diaphragmatic weakness or established hypoventilation. Furthermore, specific protocols for NMDs recommend that the BiPAP device include a backup respiratory rate, a crucial safety feature in these patients. 6 9 27

Non-invasive ventilation for patients with NMDs is not just symptomatic therapy; it is an intervention with the potential to modify the course of the disease and prolong life. In diseases such as ALS, NIV improves survival. The interaction between OSA and neuromuscular diseases is a complex field that necessitates a thorough understanding of clinical aspects. The unique pathophysiology, driven by progressive respiratory muscle weakness, distinguishes these patients from those with OSA in the general population. 9 27

Ambrosino et al. (2009) 1 demonstrated that patients who initiated NIV before the onset of daytime symptoms, hypoventilation, daytime hypercapnia, and FVC < 65% had improved quality of life, increased life expectancy after diagnosis of NMD, and prolonged tracheostomy-free survival. 1 Our study demonstrated that the indication for NIV was based on a combination of spirometric and/or polysomnographic criteria. It was observed that a significant proportion of patients initiated therapy based on FVC and/or AHI criteria, highlighting the relevance of sleep apnea in clinical decision-making for this population.

Our main limitations for the current study were the lack of blood gas analysis before, during, or after the home polygraphy due to budgetary constraints, as well as data on the degree of muscle weakness, functional scales, and disease progression, which might limit interpretation. Moreover, it is important to clarify that the present study does not aim to validate a method for early diagnosis, but to emphasize the importance of these evaluations in clinical practice.

**Figure 1:**
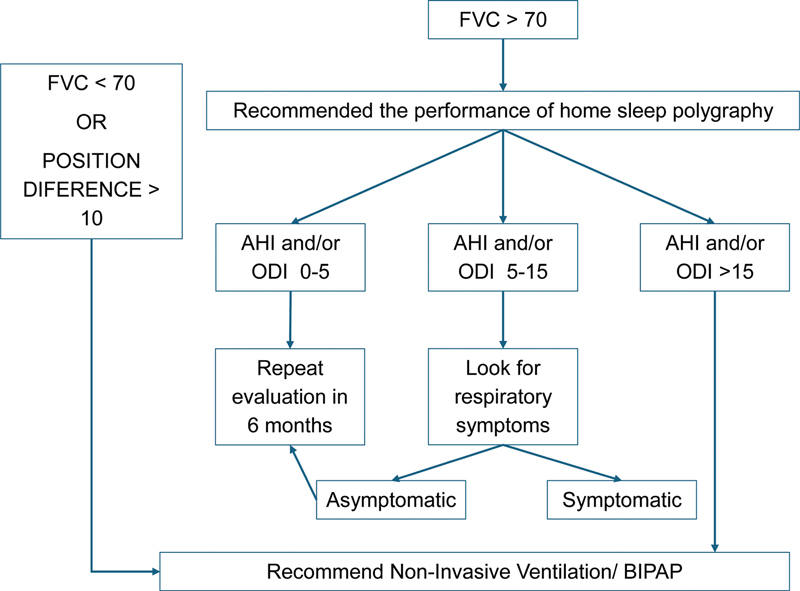
Flowchart for the evaluation of neuromuscular disease patients.

In conclusion, this study's results reinforce the importance of early diagnosis of obstructive sleep apnea in patients with neuromuscular diseases, given the significant impact of sleep disorders. Furthermore, we emphasize the need for additional epidemiological studies to investigate the relationship between various neuromuscular diseases and sleep disorders, thereby expanding our knowledge and informing more effective diagnostic and therapeutic strategies. Based on the collected data, we propose a clinical and laboratory follow-up flowchart for NMD patients at risk of obstructive and/or restrictive impairment. This flowchart aims to enable early recognition of these conditions and facilitate timely referral for home NIV in this population, as illustrated in Figure 1 .
